# Number of prior episodes and the presence of depressive symptoms are associated with longer length of stay for patients with acute manic episodes

**DOI:** 10.1186/1744-859X-11-7

**Published:** 2012-03-10

**Authors:** Manuel Martin-Carrasco, Ana Gonzalez-Pinto, Jaime L Galan, Javier Ballesteros, Jorge Maurino, Eduard Vieta

**Affiliations:** 1 Instituto de Investigaciones Psiquiátricas (Fundación Mª Josefa Recio), CIBERSAM, Pamplona, Spain; 2 Department of Psychiatry, Hospital Santiago Apóstol, University of the Basque Country, CIBERSAM, Vitoria, Spain; 3 Hospital Quirón, Málaga, Spain; 4 Department of Neuroscience-Psychiatry, University of the Basque Country, CIBERSAM, Leioa, Spain; 5 AstraZeneca Medical Department, Madrid, Spain; 6 Bipolar Disorders Programme, Hospital Clínic, University of Barcelona, IDIBAPS, CIBERSAM, Barcelona, Spain

**Keywords:** bipolar disorder, depressive symptoms, length of stay, mania

## Abstract

**Background:**

Few studies have analyzed predictors of length of stay (LOS) in patients admitted due to acute bipolar manic episodes. The purpose of the present study was to estimate LOS and to determine the potential sociodemographic and clinical risk factors associated with a longer hospitalization. Such information could be useful to identify those patients at high risk for long LOS and to allocate them to special treatments, with the aim of optimizing their hospital management.

**Methods:**

This was a cross-sectional study recruiting adult patients with a diagnosis of bipolar disorder (*Diagnostic and Statistical Manual of Mental Disorders*, 4th edition, text revision (DSM-IV-TR) criteria) who had been hospitalized due to an acute manic episode with a Young Mania Rating Scale total score greater than 20. Bivariate correlational and multiple linear regression analyses were performed to identify independent predictors of LOS.

**Results:**

A total of 235 patients from 44 centers were included in the study. The only factors that were significantly associated to LOS in the regression model were the number of previous episodes and the Montgomery-Åsberg Depression Rating Scale (MADRS) total score at admission (*P *< 0.05).

**Conclusions:**

Patients with a high number of previous episodes and those with depressive symptoms during mania are more likely to stay longer in hospital. Patients with severe depressive symptoms may have a more severe or treatment-resistant course of the acute bipolar manic episode.

## Background

According to epidemiological studies, the estimated lifetime prevalence of bipolar disorder in Europe is approximately 1% [1,2]. However, the prevalence and incidence rates are probably underestimated. A Spanish study found that only 30% of patients received a bipolar disorder diagnosis on first evaluation [3] and misdiagnosis is especially frequent in young people [4]. Bipolar disorder is often associated with other coexisting morbidities, the most common being anxiety and substance use disorders [5-7], which are related to an increased risk of suicidal ideation [8] and of mood switches from depression to mania [9,10].

In the 15 to 44 age group, bipolar disorder is among the leading causes of disability in the developed world [11], which partially explains the important economic burden of the disease. Additionally, bipolar disorder patients use healthcare services more than patients with other mental disorders [12]. Hospital admission is generally required in acute manic episodes, which are difficult to treat in outpatient settings due to insufficient adherence to treatment, and also in episodes with severe depressive symptoms and increased suicide risk [13]. The costs associated with the hospitalization of patients during acute episodes represent the largest share of the direct costs of bipolar disorders [14]. In this context, any intervention that could reduce the need for hospitalization would have a significant impact on the costs associated with the disease [15].

Length of stay (LOS) has been reported to be an important factor for determining the relative cost effectiveness of treatments for bipolar disorder [16]. Therefore, managing the cost of healthcare related to this illness requires better information regarding the factors contributing to LOS. Several studies have identified different sociodemographic characteristics as predictors for longer LOS: age, history of commitment, recent employment history, accommodations problems, and living in areas lacking community services [17-22]. Tulloch *et al. *recently published a systematic review on the length of stay of general psychiatric inpatients in the USA. Psychosis, female gender and larger hospital size were associated with longer LOS [23]. In bipolar patients, mixed episodes [13] and the Young Mania Rating Scale (YMRS) insight and language/thought disorder subscores [24] are some of the factors that have also been associated with longer LOS. Few studies have analyzed the relative contribution of diagnosis, demographic and social data and severity of illness in predicting LOS in patients hospitalized due to bipolar mania. Such information could be useful for identifying those patients at high risk for long LOS and for allocating them to special treatments, with the aim of optimizing their hospital management and achieving better clinical outcomes.

The aim of this study was to estimate LOS in patients admitted due to acute bipolar manic episodes and to determine the potential demographic and clinical risk factors associated with a longer LOS in this group of patients.

## Methods

### Study design

A multicenter, cross-sectional study was carried out in hospitalized patients with acute manic episodes. The study was conducted in 44 centers throughout Spain from November 2007 to July 2008. Mental health services are primary funded from taxes in Spain, and LOS is free of charge [25].

All patients provided written informed consent to participate in the study. The study was conducted in accordance with the ethical principles of the Declaration of Helsinki and was reviewed and approved by the Ethics Committee of the Hospital Clinico San Carlos (Madrid, Spain).

### Patients

The specific inclusion criteria were: adults aged 18 to 65 years; a bipolar disorder diagnosis based on the *Diagnostic and Statistical Manual of Mental Disorders*, 4th edition, text revision (DSM-IV-TR); hospitalization admission due to an acute manic episode (as defined by the DSM-IV-TR); total YMRS [26] score > 20; and patient ability to understand and comply with the requirements of the study. Exclusion criteria were: mental retardation, manic episode associated to drug use according to the investigator criterion, participation in a clinical trial with drugs within the 30 days prior to the inclusion in the study, and any clinical contraindication considered by the investigator.

### Assessments

Sociodemographic and illness history data were collected from medical records and clinical interviews. Psychometric evaluations were made within the first 24 h following the hospital admission and included the following scales to assess the intensity of mania and coexisting anxiety and depression symptoms: YMRS [26], the Hamilton Anxiety Rating Scale (HARS) [27], the Hamilton Depression Rating Scale 5 items (HDRS5) [28], the Montgomery-Åsberg Depression Rating Scale (MADRS) [27], and the modified Clinical Global Impression - Bipolar Disorder severity of illness scale (CGI-BP) [29]. The CGI-BP consists of three subscales providing an overall illness score and scores for manic (CGI-BP-M) and depressive symptoms (CGI-BP-D). Additionally, the general items of the Scale of Unawareness of Mental Disorders (SUMD) [30] were used to assess insight.

### Statistical analysis

LOS was defined as the total number of days a patient spent as full-time inpatient. Since LOS distribution was positively skewed (Shapiro-Wilk W test *P *< 0.01), no parametric tests (Spearman's correlation, Mann Whitney U test, Kruskal-Wallis test) were used to evaluate the unadjusted association between independent variables and LOS. The following variables were considered in the univariate analyses: sociodemographic variables (sex, age, cohabitation, and educational level), clinical factors (polarity of the onset episode, number and polarity of previous episodes, number of previous hospitalizations, presence of suicidal ideation, number of suicide attempts and family history of psychiatric disorders) and psychometric evaluations.

Only those variables with *P *≤ 0.2 in the univariate analysis were included in a Cox regression analysis with backward elimination method to identify whether they were independent predictors of LOS. The selection of survival analysis instead of linear models is based on previous studies that have used this time-to-event analysis to predict LOS in a variety of disease conditions. The correlation matrix for the independent variables was examined to detect potential multicolinearity problems in model building and to select the final candidate predictors to be included in the model based on clinical criteria. Additionally, a multiple regression analysis was performed to identify independent predictors of mania severity in this group of patients. Data analysis was performed using SAS V.8.02 (SAS, Cary, NC, USA).

## Results

We recruited 235 patients, 102 male and 133 female. The mean (SD) age was 43.0 (12) years (range, 18 to 65 years). Most patients (n = 152; 64.7%) had a manic episode as the first recognized bipolar disorder episode. A total of 185 patients (78.7%) presented past depressive episodes, 193 (82.1%) manic episodes, 65 (27.7%) hypomanic episodes and only 51 (21.7%) mixed episodes. The mean numbers of lifetime prior manic and depressive episodes of bipolar disorder were 3.9 (4.5) and 2.4 (3.0), respectively. Of the 235 patients, the index admission was the first for 47 patients (20%). Table 1 summarizes the main sociodemographic and clinical characteristics of the sample.

**Table 1:** Demographic and clinical characteristics of the sample

Variable	Value
Age in years, mean (SD)	43.0 (12.0)
Female gender, N (%)	133 (56.9)
Living alone, N (%)	38 (16.4)
Primary/secondary education only, N (%)	187 (79.5)
Polarity of the first episode, N (%):	
Depressive	74 (31.5)
Manic	152 (64.7)
Hypomanic	5 (2.1)
Mixed	4 (1.7)
Suicide attempts, N (%)	59 (25.1)
Family history of psychiatric disorders, N (%)	107 (45.5)
YMRS score, mean (SD)	33.3 (6.9)
MADRS score, mean (SD)	14.8 (7.0)
HARS score, mean (SD)	14.3 (7.1)
CGI-BP overall score, mean (SD)	4.4 (1.4)
CGI-BP mania score, mean (SD)	5.2 (0.8)
CGI-BP depression score, mean (SD)	2.0 (1.1)
SUMD overall score, mean (SD)	9.5 (3.5)

CGI-BP = Clinical Global Impression - Bipolar Disorder Scale; HARS = Hamilton Anxiety Rating Scale; MADRS = Montgomery-Åsberg Depression Rating Scale; SUMD = Scale to Assess Unawareness of Mental Disorder; YMRS = Young Mania Rating Scale.

The final sample for the LOS analysis comprised 214 admissions (21 patients were excluded because length of hospitalization was not available). The median LOS in this group of patients was 18 days (range, 1 to 73 days). There were no differences in LOS according to any of the sociodemographic variables considered (sex, age and cohabitation). The number of previous episodes was the only psychiatric history variable that seemed to be correlated with LOS in the univariate analysis (*P *≤ 0.2). All the psychometric variables had a *P *≤ 0.2 in the univariate analyses, however some of them were not included in the final model due to their high correlation (r > 0.5) with other variables selected based on clinical criteria. Thus, while YMRS score was highly correlated with CGI-BP-M score, MADRS score was highly correlated with HDRS, HARS and CGI-BP-D scores (Table 2).

**Table 2:** Correlation matrix among psychometric variables

	YMRS	HARS	HDRS5	SUMD	CGI-BP-D	CGI-BP-M	CGI-BP	MADRS
YMRS	1.000	0.215**	0.043	0.397**	-0.114	0.532**	0.250**	0.143**
HARS		1.000	0.646**	-0.076	0.486**	0.107	0.155*	0.665**
HDRS5			1.000	-0.209**	0.677**	-0.015	0.091	0.712**
SUMD				1.000	-0.310**	0.294**	0.200**	-0.162
CGI-BP-D					1.000	-0.033	0.206**	0.680**
CGI-BP-M					-	1.000	0.403**	0.055
CGI-BP							1.000	0.292**
MADRS								1.000

Significant correlation coefficients > 0.5 are shown in bold.

*P < 0.05, **P < 0.01.

CGI-BP = Clinical Global Impression - Bipolar Disorder Scale; CGI-BP-D = Clinical Global Impression-Bipolar Disorder Scale Depression; CGI-BP-M = Clinical Global Impression-Bipolar Disorder Scale Mania HARS = Hamilton Anxiety Rating Scale; HDRS5 = Hamilton Depression Rating Scale 5 items; MADRS = Montgomery-Åsberg Depression Rating Scale; SUMD = Scale to Assess Unawareness of Mental Disorder; YMRS = Young Mania Rating Scale.

Five independent variables were included in the Cox regression model based on their likely association to LOS according to univariate analyses results: the number of previous episodes (both depressive and maniac), YMRS, SUMD, MADRS and CGI-BP scores. These variables were positively correlated to LOS as it is shown in Table 3. The only factors that were significantly associated with LOS in the Cox regression model were the number of previous episodes and MADRS score within the first 24 h following the hospital admission (*P *= 0.001 and *P *= 0.030, respectively) (Table 4). Both factors decreased the probability of discharge (that is, a longer length of stay) (HR < 1.0).

**Table 3:** Spearman's correlation coefficients of variables included in the Cox regression model

	Spearman's correlation	*P *value
Number of previous episodes	0.115	0.093
YMRS score	0.206	0.003
SUMD score	0.114	0.096
CGI-BP score	0.125	0.073
MADRS score	0.185	0.007

CGI-BP = Clinical Global Impression - Bipolar Disorder Scale; MADRS = Montgomery-Åsberg Depression Rating Scale; SUMD = Scale to Assess Unawareness of Mental Disorder; YMRS = Young Mania Rating Scale.

**Table 4:** Independent predictors of LOS in the multivariate Cox regression model

Predictors	Hazard ratio	95% CI	*P *value
Number of previous episodes	0.978	0.959 to 0.997	0.021
MADRS score	0.981	0.962 to 1.000	0.045

MADRS = Montgomery-Åsberg Depression Rating Scale; LOS = length of stay.

Correlation between MADRS individual item scores and LOS were calculated in order to evaluate which items might contribute more to predict LOS. The score of two items (concentration difficulties and inability to feel) were significantly positively correlated with LOS (r = 0.273 and r = 0.241, respectively; *P *< 0.05).

Severity of mania assessed with the YMRS scale was not a significant predictor of LOS according to the results of the Cox regression analysis. The effect of YMRS score on LOS might be obscured by other factors included in the analysis that were also related to mania severity. In order to clarify this point, a multivariate linear regression analysis was performed to identify independent predictors of mania severity in this group of patients. As in the Cox regression model, a preliminary univariate analysis including the 235 patients allowed to select those sociodemographic, clinical and psychometric variables to be included as likely covariates of the final model. To rule out circularity effects, potential direct correlates of YMRS, such as CGI-BP-M, were not used. The results of the multivariate linear regression analysis indicated that YMRS was significantly explained by SUMD and MADRS scores (*P *< 0.01). While SUMD score explained approximately 15% (*R^2 ^*= 0.149) of the variation, MADRS score explained only the 4.8% (*R^2 ^*= 0.048). Two items of the MADRS, reduced sleep and concentration difficulties, were significantly positively correlated with YMRS score (r = 0.316 and r = 0.280, respectively; *P *< 0.05).

## Discussion

The main finding of this study was that patients with higher score on the MADRS scale and/or a larger number of previous episodes were more likely to remain hospitalized for longer periods of time. Therefore, those patients with severe depressive symptoms might also have a more severe or treatment-resistant course of the acute bipolar manic episode. This result highlights the importance of evaluating depressive symptoms in hospitalized patients with acute manic episodes.

Our results are consistent with evidence that hospitalizations for mixed episodes are longer than hospitalizations for depressive and manic episodes [13,31,32]. Ösby *et al. *observed that LOS was not different between manic (29.2 days) or depressive episodes (29.9 days), but was longer for mixed episodes (42.3 days) [13]. Another study carried out in Spain also found some differences in LOS according to the type of episode: 17.5, 18.4 and 21.9 days for manic, depressive and mixed episodes, respectively [31]. While in these studies a categorical assessment of the polarity of the episodes was used, in our study we used a dimensional perspective that yielded similar results.

These data are clinically significant and should stimulate further research in the field of depression monitoring and treatment during manic episodes. In addition to the economic impact of prolonged hospitalization in patients with bipolar disorders, longer LOS could have also a deleterious effect on the outcome of the patients or undermine the benefits of treatment.

Patients with more previous episodes may be more likely to stay longer in the hospital because they may have higher propensity to switch and fluctuate during hospitalization than patients with fewer episodes. Moreover, number of previous episodes has been consistently associated with severity of illness and lack of response in the current episode, allowing a longer stay in hospital [33,34].

Nowadays, the majority of patients are treated in outpatient settings and LOS in general hospital psychiatric units is measured in days [18]. However, the cost of hospitalization still represents the most significant portion of the direct costs of bipolar disorder. The mean direct cost associated with healthcare resource utilization during the manic episode was of nearly €4,500 per patient in Spain, 55% of this amount corresponding to the cost of hospitalization [15]. The costs of mania have been shown to be quite similar in different European countries [32]. Our study found patients with depressive symptoms during a manic episode are more likely to stay longer. Managing the costs associated with bipolar disorder patient care is critical due to worldwide budget containment measures. Any drug intervention in the management of mixed episodes that reduces the severity of both manic and depressive symptoms could have a significant impact on the costs associated with the disease. In addition, according to our results, mixed episodes should be reimbursed at different rates than pure manic or depressive episodes.

Overall, severity of mania measured with the YMRS scale was not a significant predictor of LOS according to our results. However, a previous study carried out in adolescents hospitalized for manic o mixed episodes found that only YMRS, insight and language/thought disorder subscores to predict longer LOS [24]. The inclusion of the MADRS score in our analysis may explain our results. The MADRS item 'inability of feel', which was positively correlated with LOS in our study, has been considered as a core depressive symptom by some authors [35]. However, since some of the other MADRS items are susceptible to contamination by a manic state, such as sleep reduction, appetite reduction, concentration difficulties and inner tension, it could be thought that the effect of the MADRS score on LOS could be explained by the manic symptoms included in this scale. Nevertheless, the results of the multivariate linear regression analysis indicated that the MADRS score only explained 4.8% of the variation in the YMRS score. Moreover, only two items of the MADRS, reduced sleep and concentration difficulties were significantly and positively correlated to the YMRS score. The fact that reduced sleep and distractibility correlated with the YMRS score indicates that these symptoms are not specific of depression. Some of the MADRS items, such as those two, may actually be measuring symptoms that can be ascribed to a manic syndrome, but other items are unequivocally linked to depression and not mania. Most definitions of mixed states actually acknowledge that not all depressive symptoms qualify as 'core' symptoms of one pole only [36,37].

The SUMD score explained a higher variance of the YMRS (15%). Lack of insight is a core symptom during mania, represents one of the treatment targets during hospitalizations, and is highly associated to treatment non-adherence [38]. Our findings indicate that the SUMD scores correlated positively with the YMRS scores and negatively with the MADRS scores, which is consistent with the fact that manic patients are more likely to be unaware of their condition than depressed patients. However, we did not find a correlation between SUMD and LOS. These results led us to confirm the predictive and independent effect of depression symptoms on LOS in acute manic episodes.

## Limitations

A limitation of this study was the lack of assessment of additional factors related to length of stay in patients with bipolar disorder, such as hospital profile, the availability of community health centers, mean LOS of previous hospitalizations or pharmacological treatment received [23,39]. In addition, comorbidity and drug use were not assessed, which may be relevant factors affecting LOS [22,40].

## Conclusions

Our study indicates that patients with a large number of previous episodes and those with depressive symptoms during mania are more likely to stay longer in hospital and may need more intensive treatment than purely manic patients with few previous episodes. This underscores the importance of defining a better clinical approach to acute manic episodes with depressive symptoms with the ultimate goal of achieving a better clinical outcome.

## Competing interests

This study was sponsored by AstraZeneca Spain. MM-C has received honoraria for speaking for Eli Lilly, Lundbeck, Pfizer, Janssen-Cilag, Servier, Esteve, and Novartis, and he has received a research grant from Novartis. AG-P has received grant support, acted as consultant, or given presentations for the following pharmaceutical companies: Almirall, AstraZeneca, Bristol-Myers-Squibb, Otsuka, Eli Lilly, Glaxo-Smith-Kline, Janssen-Cilag, Sanofi-Aventis, Lundbeck, Novartis, Organon, Schering-Plough, Spanish Ministry of Science and Innovation, Department of Health of the Basque Government, University of the Basque Country and Pfizer. JLG was an employee of AstraZeneca Spain when the study was designed, the data were collected, and the statistical analysis was carried out. JB does not have any conflicts of interest to declare. JM is an employee of AstraZeneca Spain. EV received honoraria as member of the EMBLEM Advisory Board from Eli Lilly and Co, and has also received grants unrelated to this study, acted as consultant, or served on advisory boards for the following entities: Astra-Zeneca, Bristol-Myers-Squibb, Eli Lilly, Forest Research Institute, Glaxo-Smith-Kline, Janssen-Cilag, Jazz, Merck-Sharpe & Dohme, Novartis, Organon, Otsuka, Pfizer, Sanofi, Servier, Seventh European Framework Programme (ENBREC), Shering-Plough, Spanish Ministry of Science and Innovation, United Biosource Corporation, and Wyeth.

## Authors' contributions

MM-C, AG-P, JLG, and EV conceived the study design. JLG performed the statistical analysis. All authors made meaningful contributions to data interpretation. MM-C, AG-P, JB, EV, and JM cowrote the final draft of the manuscript. All authors read and approved the final manuscript.
